# Risk Factors of Readmission to Pediatric Intensive Care Unit Within 1 Year: A Case-Control Study

**DOI:** 10.3389/fped.2022.887885

**Published:** 2022-05-12

**Authors:** Min Ding, Chunfeng Yang, Yumei Li

**Affiliations:** Department of Pediatric Intensive Care Unit, The First Hospital of Jilin University, Changchun, China

**Keywords:** readmission, pediatric intensive care unit, risk factors, PICU, case control study

## Abstract

**Background:**

Research on pediatric intensive care unit (PICU) readmission is lacking in China. This study was conducted to describe the risk factors associated with PICU readmission within 1 year after PICU discharge.

**Methods:**

This retrospective case-control study included patients aged from 1 month to 16 years who were discharged between January 2018 and May 2020. The case group included readmitted patients with two or more PICU admissions within 1 year during the study period. The control group included survivors with only one PICU admission during the same study period, and the controls were matched on age and sex. Demographic and clinical variables were collected from the electronic administrative database. Risk factors were analyzed by univariate and multivariate analyses.

**Results:**

From January 2018 to May 2020, 2,529 patients were discharged from the PICU, and 103 (4.07%) were readmitted within 1 year. In the univariate analysis, PICU readmission within 1 year was associated with lower weight, the presence of chronic conditions, a higher StrongKids score on admission, length of PICU stay of more than 2 weeks, the presence of dysfunction at discharge, sedation medications use, vasopressor use, and invasive mechanical ventilation in the first PICU stay. Patients had a higher StrongKids score as a surrogate for increased risk of malnutrition. In the multivariate analysis, the factors most significantly associated with PICU readmission within 1 year were the presence of chronic conditions, a higher StrongKids score on admission, and length of PICU stay of more than 2 weeks in the first PICU stay. In the subgroup analysis, compared with the control group, the factors most significantly associated with readmission within 48 h of discharge were the presence of chronic conditions, a higher StrongKids score on admission, and vasopressor use during the first PICU stay. The mortality rate was 8.74% (9/103) in patients with PICU readmission. The overall PICU mortality rate was 7.39% (201/2,721) during the study period.

**Conclusions:**

Patients with chronic conditions, a higher StrongKids score on admission, and length of PICU stay of more than 2 weeks were at much higher risk for PICU readmission within 1 year. Patients with vasopressor use during the first PICU hospitalization were more likely to be readmitted within 48 h of discharge.

## Background

Advances in critical care medicine have significantly improved the survival of critically ill patients worldwide. However, some studies have reported the increasing frequency and mortality of patients readmitted to the pediatric intensive care unit (PICU) (1, 2). Readmission would increase strain on health care resources and cost-effectiveness. Repeated critical illness and hospitalization also negatively impact patients and families. A better understanding of the frequency, risk factors, and outcomes of readmission will help guide interventions to reduce unnecessary readmission and potentially improve patient outcomes.

Currently, the research on PICU readmission is mostly concentrated in developed countries, and there is no generalizable information regarding PICU readmission in China. Only a few studies have been conducted to identify the risk factors associated with PICU readmission. The PICU readmission rates in those studies ranged between 1.6 and 60%, and the children had a mortality rate of 2–21.3%, which was higher than the overall mortality in the PICU (3–11%) (1–6). Most international studies have focused on exploring the early readmission within 48 or 72 h of discharge from the PICU (1–3, 5), and those studies tend to be based on specific patient populations such as patients in the pediatric cardiac intensive care unit and patients with tracheostomy placement, asthma, or neuromuscular disorders (3, 7–9). However, patients were more likely to have the late PICU readmission past 48 h of PICU discharge, with an incidence of 53–75.4% (10–12). Survivors of critical illness may experience impairments that can last for weeks to years, causing adverse long-term outcomes (13). Late readmission to PICU is one of the long-term adverse outcomes, we know little about its occurrence, risk factors, and outcomes in children. Only by acknowledging it will we be able to prevent and reduce harmful effects. Therefore, it is also of great significance to investigate the risk factors affecting late readmission to PICU within weeks or months of discharge.

We found only two studies that have attempted to determine the risk factors for PICU readmission within 1 year of PICU discharge (6, 14). Although the risk factors reported in those two studies were different, both studies reported that patients with chronic conditions were more likely to have PICU readmission (6, 14). Moreover, another study demonstrated that patients with chronic conditions were more likely to experience high malnutrition risk (15). The malnutrition risk is associated with adverse clinical outcomes, including longer lengths of stay and higher mortality rates (16). However, few studies are exploring the relationship between malnutrition risk and PICU readmission in children.

Therefore, in this study, we investigated the occurrence rate and outcomes of PICU readmission and determined the risk factors associated with PICU readmission within 1 year of PICU discharge.

## Methods

### Study Design and Population

This retrospective case-control study was conducted in a twenty-four-bed medical-surgical PICU of a public, university-affiliated tertiary care hospital. Each bed has a ventilator for critically ill patients who require mechanical ventilation. Our population is predominantly composed of children with medical conditions, although patients undergoing planned or emergency surgical procedures (e.g., neurosurgical, cardiac, thoracic surgical), and trauma are also admitted to the PICU. We reviewed the electronic administrative database of all patients aged 1 month to 16 years discharged from the PICU between January 2018 and May 2020. Those who died in their first PICU admission and lacked adequate clinical information were excluded. Planned admissions (e.g., planned surgery, elective follow-up procedures, admissions for procedures) were excluded. The case group included readmitted patients with two or more PICU admissions during different hospitalizations within 1 year during the study period. The control group included survivors with only one PICU admission during the same study period, and the controls were matched on age and sex. Early readmission was defined as being readmitted within 48 h of discharge from the PICU, and late readmission was defined as being readmitted past 48 h of PICU discharge. This definition was suggested by the Society of Critical Care Medicine (17). Pediatric intensivists would make decisions about PICU discharge and admission. The Institutional Review Board approved this study, with the need for informed consent waived.

### Data Collection and Validation

PICU admission and readmission statuses were confirmed through the electronic administrative database. Demographic and clinical characteristics of each patient were collected. Variables, including gender, age, weight, the StrongKids score, the Pediatric Logistic Organ Dysfunction 2 score (PELOD 2 score), the presence of chronic conditions, admission primary diagnostic category, use of invasive mechanical ventilation (yes/no), use of sedative medications (yes/no), use of vasopressors (yes/no), use of corticosteroids (yes/no), use of continuous renal replacement therapy (CRRT) (yes/no), the Functional Status Scale (FSS) score at discharge, length of stay (LOS) in the PICU, and mortality and the time interval between discharge from the PICU and the second admission were collected through a structured proforma.

StrongKids is used to evaluate malnutrition risk in children on admission (18). Each item is assigned a score that when added indicates the presence of nutrition risk as follows: 0 points, low risk; 1–3 points, medium risk; and 4–5 points, high risk. As a proxy for the severity of illness, a predicted probability of mortality was estimated using the PELOD 2 score (19). Chronic conditions were defined using Feudtner's definition and further examined in the PICU population as described by Edwards (20, 21). Chronic conditions were grouped into nine organ systems (cardiovascular, respiratory, neuromuscular, congenital/genetic, abnormalities, oncologic, metabolic/endocrinologic, renal, gastrointestinal, and hematologic/immunologic) and a tenth miscellaneous subcategory (rheumatologic, orthopedic, and psychiatric conditions). The FSS evaluates the overall functional status of pediatric patients at discharge from the PICU (22). The FSS scores range from 1 (normal function) to 5 (very severe dysfunction). Scores of 2, 3, and 4 indicate mild, moderate, and severe dysfunction, respectively. We chose 2 weeks of PICU LOS as a cut-off. This time duration has been used in the validation of risk factors for readmissions among PICU patients (23). Data on the final study subjects were verified through an extensive check of data validity by two experienced pediatric intensivists.

### Statistical Analysis

The Statistical Package for Social Sciences version 22 was used for data analysis. *p* < 0.05 was considered statistically significant. Continuous variables required normality testing at first, followed by presentation as median and interquartile ranges (IQRs). Percentage and frequency were calculated for categorical or grade variables. Variables of case and control groups were compared using the Wilcoxon signed-rank test for continuous or grade variables and the chi-square test for categorical variables. Factors that were not statistically significant in the univariate analysis were excluded in the multivariate analysis. Multivariate logistic regression analyses were performed to identify factors that significantly correlated with PICU readmission. Odds ratios (ORs) for readmission were calculated in multivariate analyses with corresponding 95% confidence intervals (CIs).

## Results

**Figure 1:**
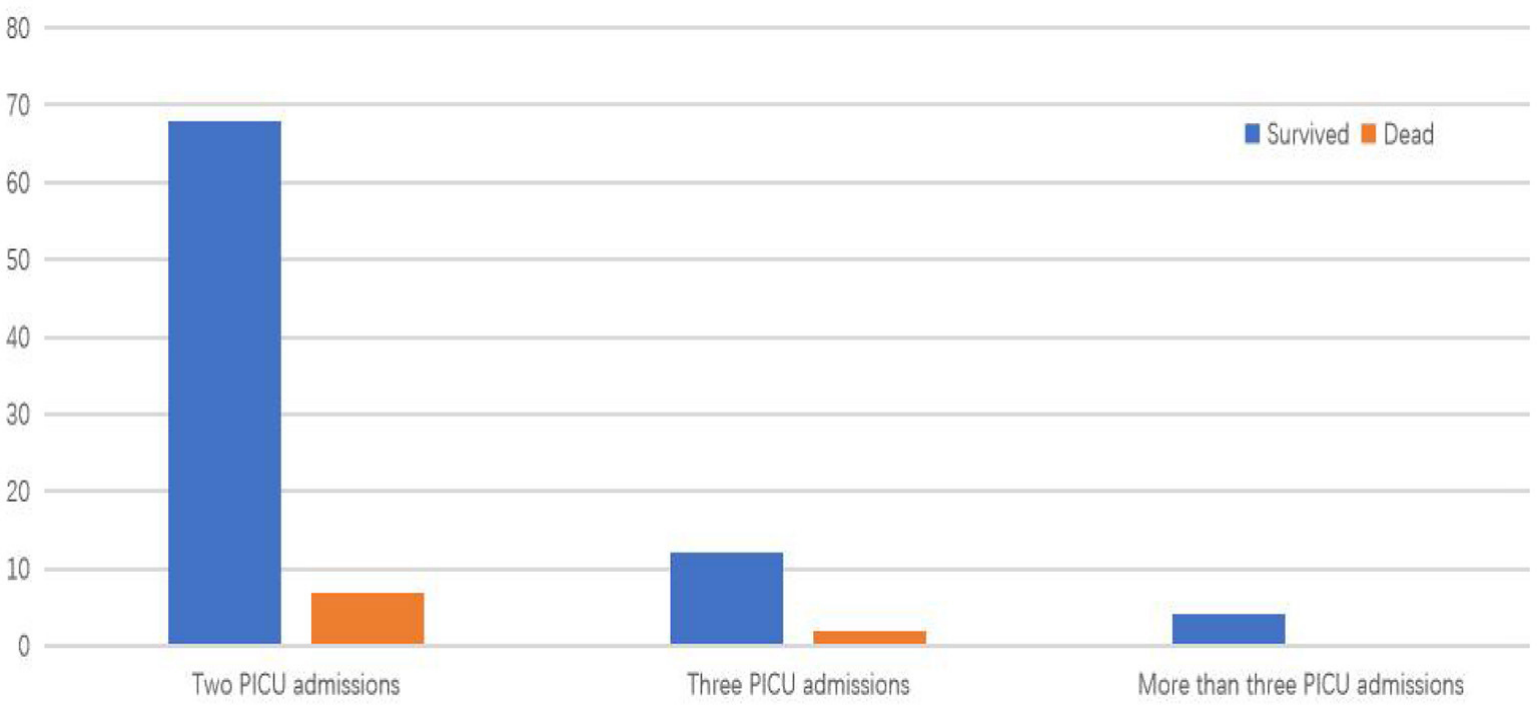
The distribution of PICU admissions in the case group.

During the study period, 2,529 patients were discharged from the PICU. The distribution of PICU admissions in the case group is shown in Figure 1. In total, 103 (4.07%) patients had PICU readmissions within 1 year of PICU discharge during the study period, of whom 85 (3.36%) had one readmission, 14 (0.55%) had two readmissions, and 4 (0.16%) had three or more readmissions. Among children with PICU readmissions, 9 patients (8.7%, 103/2,529) died, including 7 in the second PICU admission and 2 in the third PICU admission. The PICU mortality rate was 7.39% (201/2,721).

### Characteristics of Participants

Table 1 shows the characteristics of the participants. The median age was 1 year (IQR 0.42–3 years), the median weight was 10.50 kg (IQR 6–15.50 kg), and the male-to-female ratio was 1.15:1. Chronic conditions were present in 48 patients (46.60%), of whom 12 (25%) had two or more chronic conditions, with the most common chronic condition being congenital heart disease. The median StrongKids score of readmitted patients was 3 (IQR 2–3), which was higher than that of patients in the control group. The most common diagnostic category for readmission was respiratory (40.78%, *n* = 42), followed by the infection that was observed in 15 (14.56%) patients. Neurologic and cardiovascular diagnoses were found in 12 readmitted patients (11.65%). Invasive mechanical ventilation was required in 54 (52.40%) readmitted patients, and 25 patients (24.3%) required CRRT. Vasopressors were required in 49 (47.60%) readmitted patients. In the case group, 37.86% of patients were dysfunctional at discharge. More than half of readmitted patients had the first PICU LOS of more than 2 weeks.

**Table 1:** Risk factors of PICU readmission within 1 year associated with characteristics on first PICU admission.

**First PICU admission of characteristics**	**Case group** **(*N* = 103)**	**Control group** **(*N* = 206)**	***p***
Age (y), median (IQR)	1 (0.42–3.00)	1.25 (0.55-4.62)	0.076
Male sex, *n* (%)	55 (53.40)	111 (53.88)	0.819
Weight (kg), median (IQR)	10.50 (6–15.50)	11.65 (7.59–18.25)	0.048*
With chronic conditions^a^, *n* (%)	48 (46.60)	32 (15.53)	<0.001*
≥2 chronic conditions, *n* (%)	12 (25)	4 (12.50)	
With congenital heart disease, *n* (%)	10 (9.71)	12 (5.83)	0.029*
Ventricular septal defect, *n* (%)	7 (70)	6 (50)	
≥2 defects, *n* (%)	6 (60)	2 (16.67)	
Strongkids score on admission, median (IQR)	3 (2–3)	2 (1–2)	<0.001*
Admission primary diagnostic category, *n* (%)			0.127
Respiratory	42 (40.78)	89 (43.20)	
Neurologic	12 (11.65)	19 (9.22)	
Cardiovascular	12 (11.65)	17 (8.25)	
Infectious	15 (14.56)	25 (12.14)	
Trauma	7 (6.80)	22 (10.68)	
Tumor	5 (4.85)	3 (1.46)	
Hemorrhage/coagulopathy	3 (2.91)	11 (5.34)	
Urinary	3 (2.91)	9 (4.37)	
Other	4 (3.80)	11 (5.33)	
Sedation medications, *n* (%)	64 (62.14)	99 (48.05)	0.047*
Vasopressors, *n* (%)	49 (47.57)	49 (23.79)	<0.001*
Corticosteroids, *n* (%)	44 (42.72)	67 (32.52)	0.118
Invasive mechanical ventilation, *n* (%)	54 (52.43)	60 (29.12)	<0.001*
Continuous renal replacement therapy, *n* (%)	25 (24.27)	35 (16.99)	0.151
PELOD 2 score, median (IQR)	6.01 (4.24–8.25)	5.12 (2.22–6.13)	0.138
First PICU length of stay in weeks, *n* (%)			0.001*
<2	47 (45.63)	144 (69.90)	
≥2	56 (54.37)	62 (30.10)	
FSS at discharge, *n* (%)			0.007*
Normal function	64 (62.14)	165 (80.10)	
Mild dysfunction	20 (19.42)	20 (9.71)	
Moderate dysfunction	10 (9.71)	10 (4.85)	
Severe dysfunction	8 (7.77)	9 (4.37)	
Very severe dysfunction	1 (0.96)	2 (0.97)	

*IQR, interquartile range; PELOD 2 score, Pediatric Logistic Organ Dysfunction 2 score; FSS, Functional Status Scale*.

*^a^Chronic conditions included neuromuscular (status epilepticus, peripheral neuropathy, birth asphyxia, intrauterine hypoxia, anoxic brain damage, Ischemic encephalopathy, developmental delay, autism), respiratory (pulmonary fibrosis, asthma), cardiovascular (supraventricular tachycardia, ventricular tachycardia, ventricular septal defect, atrial septal defect, tricuspid valve, aortic valve insufficiency/regurgitation, mitral valve insufficiency/regurgitation, patent ductus arteriosus, pulmonary hypertension, hypertension), gastrointestinal (inguinal hernia, abdominal and intestinal hernia, umbilical hernia), oncologic (hepatocellular carcinoma, ganglioneuroma), rheumatologic (systemic lupus erythematosus, thrombotic microangiopathy, thrombotic thrombocytopenic purpura, systemic sclerosis), endocrinologic (pituitary disorders), hematologic (thrombocytopenic purpura)*.

*Significant risk factor in the multivariable analysis (p < 0.05).

The characteristics of the readmitted patients between the first and second PICU admissions are shown in Supplementary Material. In the case group, patients on the second PICU admission were older but weighed less than those on the first PICU admission. The second PICU admission in the case group had lower severity of illness than the first PICU admission [3.44 (1.25–5.25) vs. 6.01 (4.24–8.25)], and fewer patients required invasive mechanical ventilation, CRRT, sedative medications, corticosteroids, or vasopressors during the second hospitalization. Readmitted patients had shorter PICU LOS in the second PICU admission. In total, 74 patients (66.06%) had the same major diagnostic category between the first and second PICU admissions. More than half of the same major diagnostic category was respiratory (*n* = 45, 60.81%), and pneumonia was the most common diagnosis in the respiratory category (*n* = 40, 88.89%). The median time interval between the first and second PICU admissions was 6 days (IQR 1.21–25).

### Risk Factors for PICU Readmission Within 1 Year

Potential risk factors during the first admission for PICU readmission are shown in Table 1. In the univariate analysis, patients with PICU readmission were significantly weighed less than control group patients (*p* = 0.048). Children with PICU readmission were more likely to have chronic conditions than those not readmitted (*p* < 0.001). The case group was more likely to have the presence of congenital heart disease (*p* = 0.029). The StrongKids score on the first PICU admission was significantly different between the case and control groups (*p* < 0.001). Patients with PICU readmission were more likely to have longer PICU stays (*p* = 0.001). They were more likely to require vasopressors (*p* < 0.001), sedative medications (*p* = 0.047) and invasive mechanical ventilation (*p* < 0.001) during PICU stay. Although the median PELOD 2 score was higher on readmission, it was not statistically significant. Children in the case group were more likely to have dysfunction at discharge than those in the control group (*p* = 0.007). Age, sex, admission primary diagnostic category, use of corticosteroids, and use of CRRT did not meet statistical significance.

In the multivariate analysis, the variables that remained statistically significant were first PICU LOS, the StrongKids score on admission, and the presence of chronic conditions. Case group patients were more likely to have chronic conditions (*p* < 0.001, OR 4.99, CI 2.81–10.22), a higher StrongKids score on admission (*p* < 0.001, OR 4.5, CI 2.70–7.60), and PICU LOS of more than 2 weeks (*p* = 0.023, OR 2.05, CI 1.08–3.92) in the first PICU admission (Table 2).

**Table 2:** Risk factors of PICU readmission within 1 year in the multivariate analysis.

**Variable**	**Adjusted odds ratio (95% CI)**	***p***
With chronic conditions	4.99 (2.81–10.22)	<0.001*
The Strongkids score on admission	4.5 (2.70–7.60)	<0.001*
First PICU length of stay in days for more than 2 weeks	2.05 (1.08–3.92)	0.023*

*CI, confidence interval*.

*Significant risk factors in the multivariable analysis (p < 0.05).

### Subgroup Analysis: Risk Factors for Early and Late PICU Readmission

Most patients (67.96%, *n* = 70) had late readmission, whereas 32.04% (*n* = 33) had early readmission. Infection was the major cause of early readmission (75.76%). We further investigated the risk factors of early readmission and late readmission to PICU. Compared with the control group, the factors most significantly associated with early readmission were the presence of chronic conditions (*p* = 0.001, OR 5.12, CI 1.99–12.66), a higher StrongKids score on admission (*p* = 0.008, OR 2.52, CI 1.27–5.34), and vasopressor use during the first PICU stay (*p* = 0.029, OR 2.77, CI 1.06–7.55) (Tables 3, 4). Late readmission patients were more likely to have chronic conditions (*p* < 0.001, OR 4.53, CI 1.97–10.41), a higher StrongKids score on admission (*p* < 0.001, OR 3.79, CI 2.06–6.94), and PICU LOS of more than 2 weeks (*p* = 0.024, OR 2.41, CI 1.31–5.58) in the first PICU admission (Tables 5, 6). Neither demographic nor clinical characteristics of early readmission were statistically significant compared with those of late readmission.

**Table 3:** Risk factors of early readmission associated with characteristics on first PICU admission.

**First PICU admission of characteristics**	**Early readmission** **(*N =* 33)**	**Control group** **(*N =* 206)**	***p***
Age (*y*), median (IQR)	1 (0.25–2)	1.25 (0.55–4.62)	0.046*
Male sex, *n* (%)	17 (51.52)	111 (53.88)	0.726
Weight (kg), median (IQR)	10.7 (5.82–10.45)	11.65 (7.59–18.25)	0.049*
With chronic conditions, *n* (%)	11 (33.33)	32 (15.53)	<0.001*
Strongkids scores on admission, median (IQR)	2 (2–3)	2 (1–2)	<0.001*
Admission primary diagnostic category, *n* (%)			0.141
Respiratory	11 (33.33)	89 (43.20)	
Neurologic	6 (18.18)	19 (9.22)	
Cardiovascular	3 (9.09)	17 (8.25)	
Infectious	4 (12.12)	25 (12.14)	
Trauma	6 (18.18)	22 (10.68)	
Tumor	2 (6.06)	3 (1.46)	
Hemorrhage/Coagulopathy	0	11 (5.34)	
Urinary	0	9 (4.37)	
Other	1 (3.04)	11 (5.33)	
Sedation medications, *n* (%)	15 (45.45)	99 (48.05)	0.522
Vasopressors, *n* (%)	14 (42.42)	49 (23.79)	0.010*
Corticosteroids, *n* (%)	13 (39.39)	67 (32.52)	0.443
Invasive mechanical ventilation, *n* (%)	15 (45.45)	60 (29.12)	0.110
Continuous Renal Replacement Therapy, *n* (%)	6 (18.18)	35 (16.99)	0.800
PELOD 2 score, median (IQR)	5.99 (1.26–8.25)	5.12 (2.22–6.13)	0.158
First PICU length of stay in weeks, *n* (%)			0.033*
<2	16 (48.48)	144 (69.90)	
≥ 2	17 (51.52)	62 (30.10)	
FSS at discharge, *n* (%)			0.144
Normal function	22 (66.67)	165 (80.10)	
Mild dysfunction	7 (21.21)	20 (9.71)	
Moderate dysfunction	3 (9.09)	10 (4.85)	
Severe dysfunction	1 (3.03)	9 (4.37)	
Very severe dysfunction	0	2 (0.97)	

*IQR, interquartile range; PELOD 2 score, Pediatric Logistic Organ Dysfunction 2 score; FSS, Functional Status Scale*.

*Significant risk factor in the multivariable analysis (p < 0.05).

**Table 4:** Risk factors of PICU early readmission in the multivariate analysis.

**Variables**	**Adjusted odds ratio (95% CI)**	***p***
With chronic conditions	5.12 (1.99–12.66)	0.001*
The Strongkids score on admission	2.52 (1.27–5.34)	0.008*
Vasopressor use	2.77 (1.06–7.55)	0.029*

*CI, confidence interval*.

*Significant risk factor in the multivariable analysis (p < 0.05).

**Table 5:** Risk factors of late readmission associated with characteristics on first PICU admission.

**First PICU admission of characteristics**	**Late readmission** **(*N =* 70)**	**Control group** **(*N =* 206)**	***p***
Age (*y*), median (IQR)	1.25 (0.50–4.44)	1.25 (0.55–4.62)	0.100
Male sex, *n* (%)	38 (54.29)	111 (53.88)	0.927
Weight (kg), median (IQR)	10.35 (6.23–17)	11.65 (7.59–18.25)	0.099
With chronic conditions, *n* (%)	37 (52.86)	32 (15.53)	<0.001*
Strongkids scores on admission, median (IQR)	3 (2–3)	2 (1–2)	<0.001*
Admission primary diagnostic category, *n* (%)			0.021*
Respiratory	31 (44.29)	89 (43.20)	
Neurologic	6 (8.56)	19 (9.22)	
Cardiovascular	9 (12.86)	17 (8.25)	
Infectious	11 (15.71)	25 (12.14)	
Trauma	1 (1.42)	22 (10.68)	
Tumor	3 (4.29)	3 (1.46)	
Hemorrhage/coagulopathy	3 (4.29)	11 (5.34)	
Urinary	3 (4.29)	9 (4.37)	
Other	3 (4.29)	11 (5.33)	
Sedation medications, *n* (%)	46 (65.70)	99 (48.05)	0.031*
Vasopressors, *n* (%)	34 (48.57)	49 (23.79)	0.001*
Corticosteroids, *n* (%)	31 (44.3)	67 (32.52)	0.103
Invasive mechanical ventilation, *n* (%)	39 (55.71)	60 (29.12)	0.001*
Continuous renal replacement therapy, *n* (%)	19 (27.1)	35 (16.99)	0.077
PELOD 2 score, median (IQR)	6.12 (2.24–9.07)	5.12 (2.22–6.13)	0.246
First PICU length of stay in weeks, *n* (%) 0.001*		
<2	30 (42.86)	144 (69.90)	
≥ 2	40 (57.14)	62 (30.10)	
FSS at discharge, *n* (%)			0.069
Normal function	42 (60)	165 (80.10)	
Mild dysfunction	13 (18.57)	20 (9.71)	
Moderate dysfunction	7 (10)	10 (4.85)	
Severe dysfunction	7 (10)	9 (4.37)	
Very severe dysfunction	1 (1.4)	2 (0.97)	

*IQR, interquartile range; PELOD 2 score, Pediatric Logistic Organ Dysfunction 2 score; FSS, Functional Status Scale*.

*Significant risk factor in the multivariable analysis (p < 0.05).

**Table 6:** Risk factors of PICU late readmission in the multivariate analysis.

**Variables**	**Adjusted odds ratio (95% CI)**	***p***
With chronic conditions	4.53 (1.97–10.41)	<0.001*
Strongkids score on admission	3.79 (2.06–6.94)	<0.001*
First PICU length of stay in days for more than 2 weeks	2.41 (1.31–5.58)	0.024*

*CI, confidence interval*.

*Significant risk factor in the multivariable analysis (p < 0.05).

## Discussion

PICU readmission can cause significant financial burdens to families and obstacles to hospital efficiency for institutions (24, 25). Understanding the risk factors for PICU readmission may help improve the quality of care and reduce its incidence. To our knowledge, this is the first study from China to estimate the incidence, risk factors, and mortality of PICU readmission. We found that patients with chronic conditions, a higher StrongKids score on admission, and PICU LOS of more than 2 weeks were at much higher risk for PICU readmission within 1 year of PICU discharge.

Based on our single-center study, the PICU readmission rate within 1 year of PICU discharge was 4.07 and 0.71% of patients had two or more readmissions. Three international studies have described a broad range of 9.8–53% of PICU readmission rates within 1 year (6, 7, 14). A recent study from a single-center PICU in Brazil reported the readmission rate within 1 year as 9.8%, and the rate of two or more readmissions was 2.5% (6). Our PICU readmission rate was lower than the latest international data. Readmission was the factor most strongly associated with pediatric death within 1 year of PICU discharge (14). The reported mortality rates of readmitted patients are variable in international data, being 2–21.3% (5, 6). In our study, the mortality rate in readmitted patients was 8.7%, which is comparable to the literature. The overall PICU mortality rate in our study (7.39%, 201/2,721) was also comparable to the 3–11% mortality rate reported in the literature (1, 2).

Previous studies revealed that chronic conditions were associated with PICU readmission (6, 12, 14, 26). A study from Brazil reported that patients with chronic conditions would have an increased risk of readmission compared with those who had not been readmitted within 1 year of PICU discharge, and neuromuscular disease was the most common chronic condition (6). Moreover, at least 65% of patients with chronic conditions would be readmitted to the PICU within 1 year after PICU discharge (6). Edwards et al. (14) also found that patients with any chronic conditions were more likely to be readmitted than those without chronic conditions within 1 year, and hemopoietic cancers had the highest hazard for readmission. In our study, children with chronic conditions had a more than five-fold risk of PICU readmission than children without chronic conditions. Most patients were not readmitted within 1 year of PICU discharge even if they had chronic conditions in our study, which is different from the research reported in Brazil. This may be one of the reasons for the lower incidence of PICU readmission in our study than that of the study in Brazil. Congenital heart disease was the most common chronic condition in our study, which was different from the abovementioned two studies. And readmitted patients had significantly more complex cardiac defects that included more than two defects than the controls, which could explain patients with the presence of congenital heart disease had a higher risk for readmission to PICU. Our regional economy and medical level are so backward that comprehensive screening and follow-up treatment are not conducted in the perinatal and neonatal period, which may cause neglect of several children with congenital heart disease. In the future, we must strengthen the screening for congenital heart disease in newborns and the research on the pathogenesis and prevention strategies of congenital heart disease to reduce the incidence. Such at-risk children, particularly those with complex cardiac defects, should be followed up on a more frequent basis after PICU discharge to reduce the incidence of readmission.

Clinical guidelines state that all patients should be screened for malnutrition risk on admission and periodically during their hospitalization (27). Higher malnutrition risk is associated with growth impairment, longer LOS, increased hospital costs, and higher mortality (28). Although few studies have directly confirmed the relationship between malnutrition risk and PICU readmission in adults or children, it has been confirmed that higher malnutrition risk is associated with the occurrence of malnutrition and malnutrition will cause readmission (29). In recent studies of different nutrition screening tools in pediatrics, StrongKids was considered a method of evaluating good clinical performance (30). Although the median StrongKids scores of both case and control groups indicated medium risk in our study, the case group had higher StrongKids scores than the control group. Moreover, readmitted patients on the second PICU admission were older but weighed less than those on the first PICU admission. Considering the presence of chronic conditions is one of the most important factors in determining the risk of malnutrition, the increased risk of malnutrition in the case group on admission could be attributed to the presence of chronic conditions in more patients. As a result, we should pay more attention to chronic conditions management during critical care and offer adequate treatment as well as meticulous pre-discharge assessment to avoid readmissions.

PICU readmission within 48 h has been proposed as a quality metric by the Society of Critical Care Medicine in 1995 (17). In 2007, the National Quality Forum has also supported early PICU readmission as a pediatric-specific quality measure (31). Therefore, we also described the risk factors for early PICU readmission. We observed that 32.04% of PICU readmission was early readmission, which is almost similar to the literature, where the early readmission rate was <50% among PICU readmissions (10–12). The risk factors for early readmission are similar to those of late readmission compared with the control group. However, the presence of chronic conditions and the higher malnutrition risk were significantly associated with early readmission and late readmission, and patients with vasopressor use during the first PICU hospitalization were more likely to have early readmission. Although there is no similar result to our finding, a report from the pediatric cardiac intensive care unit also demonstrated that discontinuation of vasoactive infusions at ≤ 24 h before PICU discharge was associated with early readmission. Therefore, patients with vasopressor use during hospitalization may have complex medical needs, placing them at risk for early repeated admissions (3). We also found that the past 75% of patients had early PICU readmission due to infection, indicating the need to focus on nosocomial infection during the process of hospital nursing.

Most readmitted patients had the same major diagnostic category between the first and second PICU admissions in our study, and the disease severity on the second PICU admission was significantly lower than that on the first PICU admission. However, Paulo et al. found that more than half of patients had PICU readmissions within 1 year of discharge due to the new diagnostic category, and the median time interval between the first and second PICU admissions was 73 days (6). We discovered that the median time interval between first and second PICU admission was 6 days, indicating the possibility of readmission after discharge due to inadequacy of treatment and the deterioration of the patient's condition in the first PICU admission. These findings suggested that children should be fully evaluated before discharge and the hospital decision should be made carefully. We can set a pre-discharge score for discharge, and if the criterion is not met, the PICU stay can be extended if necessary. Early readmission to the PICU is defined as within 48 h in developed countries, but in developing countries or medically underdeveloped regions, 1 week may be a more appropriate time to assess the PICU quality.

Our study limitations warrant consideration. First, we had no data on whether patients were readmitted to other PICUs or death occurred outside our institution, which may lead us to underestimate the number of readmissions and deaths. However, our institution has the only separate PICU of a tertiary care university-affiliated institution in our region. Second, we had no data regarding PICU capacity strain on the day of discharge. This may affect PICU doctors' decisions regarding patients' discharge. It would be useful to have information regarding resource utilization to determine the impact of “ICU strain” on readmission.

## Conclusions

Patients with chronic conditions, a higher StrongKids score on admission, and PICU LOS of more than 2 weeks were at much higher risk for PICU readmission within 1 year. Patients with vasopressor use during PICU hospitalization were more likely to get readmitted within 48 h of discharge. We characterized the readmission rate and identified the characteristics highly associated with readmission. Better recognition of patients at higher risk of readmission may improve resource utilization by targeting closer follow-up or outreach services for these patients after their discharge from the PICU.

## Data Availability Statement

The raw data supporting the conclusions of this article will be made available by the authors, without undue reservation.

## Ethics Statement

The studies involving human participants were reviewed and approved by the First Hospital of Jilin University Institutional Review Board. Written informed consent from the participants' legal guardian/next of kin was not required to participate in this study in accordance with the national legislation and the institutional requirements.

## Author Contributions

MD: conceptualization, methodology, investigation, and writing—original draft. CY: writing—review and editing, and software. YL: project administration. All authors contributed to the intellectual content of this manuscript and approved the final manuscript as submitted.

## Funding

The study is funded by the subject named Clinical study of ultra-early rehabilitation combined with pediatric tuina to improve the prognosis of critically ill children.

## Conflict of Interest

The authors declare that the research was conducted in the absence of any commercial or financial relationships that could be construed as a potential conflict of interest.

## Publisher's Note

All claims expressed in this article are solely those of the authors and do not necessarily represent those of their affiliated organizations, or those of the publisher, the editors and the reviewers. Any product that may be evaluated in this article, or claim that may be made by its manufacturer, is not guaranteed or endorsed by the publisher.

## Supplementary Material

The characteristics of the readmitted patients between the first and second PICU admissions are shown in Supplementary Material.

## References

[1] Khan MR Maheshwari PK Iram S Haque A Kayaalp C. Readmission to paediatric intensive care unit: frequency, causes and outcome. J Coll Physicians Surg Pak. (2014) 24:216-7. 24613123

[2] Bastero-Miñón P Russell JL Humpl T. Frequency, characteristics, and outcomes of pediatric patients readmitted to the cardiac critical care unit. Intensive Care Med. (2012) 38:1352–7. 10.1007/s00134-012-2592-2 22588651

[3] Smith AH Anand V Banerjee M Bates KE Brunetti MA Cooper DS. Variation in case-mix adjusted unplanned pediatric cardiac ICU readmission rates. Crit Care Med. (2018) 46:e1175–82. 10.1097/CCM.0000000000003440 30252712 PMC6239958

[4] Chacko JJ Pidborochynski T Buchholz H Freed DH Al-Aklabi M Anand V. Discharge and readmission to the pediatric cardiac icu in pediatric patients with durable ventricular assist devices. Pediatr Crit Care Med. (2020) 21:e810–8. 10.1097/PCC.0000000000002456 32769703

[5] Kaur H Naessens JM Hanson AC Fryer K Nemergut ME Tripathi S. PROPER: Development of an early pediatric intensive care unit readmission risk prediction tool. J Intensive Care Med. (2018) 33:29–36. 10.1177/0885066616665806 27601481

[6] da Silva PSL Fonseca MCM. Which children account for repeated admissions within 1 year in a Brazilian pediatric intensive care unit? J Pediatr (Rio J). (2019) 95:559–66. 10.1016/j.jped.2018.04.009 29856945

[7] Heneghan JA Shein SL. Readmissions to the ICU among children with tracheostomies placed after cardiac arrest. Hosp Pediatr. (2019) 9:256–64. 10.1542/hpeds.2018-0269 30867193

[8] Ryan KS Son S Roddy M Siraj S McKinley SD Nakagawa TA. Pediatric asthma severity scores can distinguish suitable inpatient level of care for children admitted with status asthmaticus. J Asthma. (2021) 58:151–9. 10.1080/02770903.2019.1680998 31608716

[9] Yates K Festa M Gillis J Waters K North K. Outcome of children with neuromuscular disease admitted to paediatric intensive care. Arch Dis Child. (2004) 89:170–5. 10.1136/adc.2002.019562 14736637 PMC1719795

[10] Kalzén H Larsson B Eksborg S Lindberg L Edberg KE Frostell C. Survival after PICU admission: the impact of multiple admissions and complex chronic conditions. PLoS ONE. (2018) 13:e0193294. 10.1371/journal.pone.0193294 29621235 PMC5886395

[11] Chew L Su-Velez BM Miller JE West AN. 30-Day readmission rates, diagnoses, and risk factors following pediatric airway surgery. Int J Pediatr Otorhinolaryngol. (2020) 136:110141. 10.1016/j.ijporl.2020.110141 32554136

[12] Czaja AS Hosokawa PW Henderson WG. Unscheduled Readmissions to the PICU: Epidemiology, Risk Factors, and Variation Among Centers. Pediatr Crit Care Med. (2013) 14:571–9. 10.1097/PCC.0b013e3182917a68 23823192

[13] Inoue S Hatakeyama J Kondo Y Hifumi T Sakuramoto H Kawasaki T. Post-intensive care syndrome: its pathophysiology, prevention, and future directions. Acute Med Surg. (2019) 6:233–46. 10.1002/ams2.415 31304024 PMC6603316

[14] Edwards JD Lucas AR Boscardin WJ Dudley RA. Repeated critical illness and unplanned readmissions within 1 year to PICUs. Crit Care Med. (2017) 45:1276–84. 10.1097/CCM.0000000000002439 28708677 PMC5541898

[15] Sermet-Gaudelus I Poisson-Salomon AS Colomb V Brusset MC Mosser F Berrier F. Simple pediatric nutritional risk score to identify children at risk of malnutrition. Am J Clin Nutr. (2000) 72:64–70. 10.1093/ajcn/72.1.64 10871562

[16] Beser OF Cokugras FC Erkan T Kutlu T Yagci RV; TUHAMAR Study Group. Evaluation of malnutrition development risk in hospitalized children. Nutrition. (2018) 48:40–7. 10.1016/j.nut.2017.10.020 29469018

[17] Candidate Critical Care Quality Indicators. Society of Critical Care Medicine Quality Indicators Committee: 1995. Ananheim (Calif): Society of Critical Care Medicine (1995).

[18] Jessie M. Hulst, Henrike Zwart, Wim C Hop, Koen F M Joosten. Dutch national survey to test the STRONGkids nutritional risk screening tool in hospitalized children. Clin Nutr. (2010) 29:106–11. 10.1016/j.clnu.2009.07.006 19682776

[19] Leteurtre S Duhamel A Salleron J Grandbastien B Lacroix J Leclerc F. PELOD-2: an update of the PEdiatric logistic organ dysfunction score. Crit Care Med. (2013) 41:1761–73. 10.1097/CCM.0b013e31828a2bbd 23685639

[20] Feudtner C Christakis DA Connell FA. Pediatric deaths attributable to complex chronic conditions: a population-based study of Washington State, 1980–1997. Pediatrics. (2000) 106:205–9. 10.1542/peds.106.S1.205 10888693

[21] Edwards JD Houtrow AJ Vasilevskis EE Rehm RS Markovitz BP Graham RJ. Chronic conditions among children admitted to US PICUs: their prevalence and impact on risk for mortality and prolonged length of stay. Crit Care Med. (2012) 40:2196–203. 10.1097/CCM.0b013e31824e68cf 22564961 PMC3378726

[22] Pollack MM Holubkov R Glass P Dean JM Meert KL Zimmerman J. Functional Status Scale: new pediatric outcome measure. Pediatrics. (2009) 124:e18-28. 10.1542/peds.2008-1987 19564265 PMC3191069

[23] Hartman ME Saeed MJ Bennett T Typpo K Matos R Olsen MA. Readmission and Late Mortality After Critical Illness in Childhood. Pediatr Crit Care Med. (2017) 18:e112–21. 10.1097/PCC.0000000000001062 28107264 PMC5336515

[24] Manning JC Hemingway P Redsell SA. Long-term psychosocial impact reported by childhood critical illness survivors: A systematic review. Nurs Crit Care. (2014) 19:145–56. 10.1111/nicc.12049 24147805 PMC4285805

[25] Netzer G Sullivan DR. Recognizing, naming, and measuring a family intensive care unit syndrome. Ann Am Thorac Soc. (2014) 11:435–41. 10.1513/AnnalsATS.201309-308OT 24673699 PMC4028736

[26] Odetola FO Clark SJ Dechert RE Shanley TP. Going back for more: an evaluation of clinical outcomes and characteristics of readmissions to a pediatric intensive care unit. Pediatr Crit Care Med. (2007) 8:343347. 10.1097/01.PCC.0000269400.67463.AC 17545926

[27] Mueller C Compher C Ellen DMASPEN. clinical guidelines: nutrition screening, assessment, and intervention in adults. J Parenter Enter Nutr. (2011) 35:16–24. 10.1177/0148607110389335 21224430

[28] Joosten KF Hulst JM. Malnutrition in pediatric hospital patients: current issues. Nutrition. (2011) 27:133–7. 10.1016/j.nut.2010.06.001 20708380

[29] Gambra-Arzoz M Alonso-Cadenas JA Jiménez-Legido M López-Giménez MR Martín-Rivada Á de Los Ángeles Martínez-Ibeas M. Nutrition risk in hospitalized pediatric patients: higher complication rate and higher costs related to malnutrition. Nutr Clin Pract. (2020) 35:157–63. 10.1002/ncp.10316 31144381

[30] Teixeira AF Viana KD. Nutritional screening in hospitalized pediatric patients: a systematic review. J Pediatr (Rio J). (2016) 92:343–52. 10.1016/j.jped.2015.08.011 26859247

[31] Pleacher KM Bratton SL. Pediatric intensive care unit bounce-backs: are we evaluating them? Pediatr Crit Care Med. (2007) 8:401–2. 10.1097/01.PCC.0000269382.31969.71 17622924

