# Supplementary material for: Risk Factors of Readmission to Pediatric Intensive Care Unit Within 1 Year: A Case-Control Study
Source: Front Pediatr. 2022 May 12;10:887885. doi: 10.3389/fped.2022.887885 (PMC9133623; doi:10.3389/fped.2022.887885)
Supplement: Supplementary file 1 [file Table_1.DOC]

Table Characteristics of the first and second PICU admissions in case group.

| Characteristics | First PICU admission | Second PICU admission |
| --- | --- | --- |
| Age (y), median (IQR) | 1 (0.42-3） | 1.50 (0.50-3) |
| Weight (kg), median (IQR) | 10.50 (6-15.50) | 10.20 (6.20-16) |
| PELOD 2 score, median (IQR) | 6.01 (4.24-8.25) | 3.44 (1.25-5.25) |
| Sedation medications, n (%) | 64 (62.14) | 27 (26.20) |
| Vasopressors, n (%) | 49 (47.57) | 14 (13.60) |
| Corticosteroids, n (%) | 44 (42.72) | 27 (26.20) |
| Invasive mechanical ventilation, n (%) | 54 (52.43) | 19 (18.40) |
| Continuous renal replacement therapy, n(%) | 25 (24.27) | 10 (9.70) |
| PICU length of stay in days, median (IQR) | 14 (8-25） | 6 (4-10) |
| Same diagnostic category for first and second admission, n (%) | 74 | |
| Respiratory | 45 (60.81) | |
| Pneumonia | 40 (88.89) | |
| Neurologic | 8 (10.81) | |
| Cardiovascular | 5 (6.76) | |
| Interval time between the first and second admissions in days, median (IQR) | 6 (1.21-25) | |
| Early readmission, n (%) | 33 (32.03) | |
| Infectious admission reasons, n (%) | 25 (75.76) | |
| Late readmission, n (%) | 70 (67.96) | |

Abbreviations: IQR, interquartile range; PELOD 2 score, Pediatric Logistic Organ Dysfunction 2 score.
